# Overview of systematic reviews of therapeutic ranges: methodologies and recommendations for practice

**DOI:** 10.1186/s12874-017-0363-z

**Published:** 2017-06-02

**Authors:** Lewis Cooney, Yoon K. Loke, Su Golder, Jamie Kirkham, Andrea Jorgensen, Ian Sinha, Daniel Hawcutt

**Affiliations:** 1 Institute of Translational Medicine, University of Liverpool, Liverpool, Merseyside UK; 2 School of Medicine, Health Policy and Practice, University of East Anglia, Norwich, UK; 3 Department of Health Sciences, University of York, York, UK; 4 Department of Biostatistics, University of Liverpool, Liverpool, UK; 5 Respiratory Department, Alder Hey Children’s Hospital, Liverpool, UK; 6 National Institute for Health Research Alder Hey Clinical Research Facility, Alder Hey Children’s Hospital, Liverpool, Merseyside UK

**Keywords:** Systematic review methodology, Therapeutic range, Systematic review

## Abstract

**Background:**

Many medicines are dosed to achieve a particular therapeutic range, and monitored using therapeutic drug monitoring (TDM). The evidence base for a therapeutic range can be evaluated using systematic reviews, to ensure it continues to reflect current indications, doses, routes and formulations, as well as updated adverse effect data. There is no consensus on the optimal methodology for systematic reviews of therapeutic ranges.

**Methods:**

An overview of systematic reviews of therapeutic ranges was undertaken. The following databases were used: Cochrane Database of Systematic Reviews (CDSR), Database of Abstracts and Reviews of Effects (DARE) and MEDLINE. The published methodologies used when systematically reviewing the therapeutic range of a drug were analyzed. Step by step recommendations to optimize such systematic reviews are proposed.

**Results:**

Ten systematic reviews that investigated the correlation between serum concentrations and clinical outcomes encompassing a variety of medicines and indications were assessed. There were significant variations in the methodologies used (including the search terms used, data extraction methods, assessment of bias, and statistical analyses undertaken). Therapeutic ranges should be population and indication specific and based on clinically relevant outcomes. Recommendations for future systematic reviews based on these findings have been developed.

**Conclusion:**

Evidence based therapeutic ranges have the potential to improve TDM practice. Current systematic reviews investigating therapeutic ranges have highly variable methodologies and there is no consensus of best practice when undertaking systematic reviews in this field. These recommendations meet a need not addressed by standard protocols.

## Background

The therapeutic range of a drug is the dosage range or blood plasma or serum concentration usually expected to achieve the desired therapeutic effect. This does not mean that patients may not achieve benefit at concentrations below the minimum threshold, or may not experience adverse effects if kept within the range.

In order to maintain a patient within a defined therapeutic range, they may be subject to therapeutic drug monitoring (TDM). TDM involves measuring drug concentrations, usually in the blood, and comparing the result to a predefined window of serum concentrations that are considered to reflect the optimum efficacy and safety of the drug. As TDM is invasive, and will inform important clinical decisions, it is important that target therapeutic ranges are appropriate. If they are not, patients may not experience the full potential benefit of the drug, or could be at risk of avoidable adverse effects. High quality evidence of clinical efficacy and safety should determine a drug’s therapeutic range.

The upper and lower boundaries of the therapeutic range should be determined by the extent of harm and benefit respectively. Many drugs were assigned therapeutic ranges based on small pharmacokinetic studies performed in the 1960s and 1970s [1–3] or expert opinion. Most drugs are typically assigned a single therapeutic range for all indications, at all ages, and regardless of co-medication and co-morbidity. Following introduction of a new drug, the age range, clinical indications, route of administration and formulation may change, while the therapeutic range is not considered. In addition, as there is a lack of empirical evidence investigating the precise serum concentrations for which a drug is most effective, reference values may differ between laboratories. This is demonstrated in carbamazepine guidelines, with quoted lower limit ranges from 4 to 8 mg/l, whilst its upper limit ranges from 8 to 12 mg/l [4].

A systematic review is the most robust method for evaluating and synthesizing existing literature on the beneficial and adverse effects of healthcare interventions. Whilst the methods are well established for studies of efficacy and increasingly so, for harm [5, 6], we were not aware of a consensus on the optimum methodology for a systematic review that aims to determine upper and lower limits of the therapeutic range for a particular drug. Such systematic reviews of therapeutic ranges are an emerging and uncertain field, but potentially more important with the advent of stratified medicine and individualised treatment due to increased understanding of interindividual variability in drug response. In this study, we have undertaken a systematic review of those systematic reviews investigating the optimum therapeutic range of a drug, and detailed the methodology used. From lessons learnt and good practice examples from these studies, we propose step-by-step recommendations for designing, analysing and presenting systematic reviews of therapeutic ranges.

## Methods

We performed a systematic overview of reviews investigating either therapeutic ranges, or those that correlate serum drug concentrations with clinically relevant outcomes.

The Cochrane Database of Systematic Reviews (CDSR) and the Database of Abstracts and Reviews of Effects (DARE) were searched for relevant literature via The Cochrane Library and Centre for Reviews and Dissemination (CRD) website. In particular, DARE uses a comprehensive search strategy to capture relevant systematic reviews from other databases including MEDLINE and EMBASE, unpublished studies and was updated regularly until the 31st March 2015. This search was supplemented by directly searching the MEDLINE database for relevant studies. The search term ‘drug monitoring’ was used as both a Medical Subject Heading (MeSH) and as a free text term with OR Boolean operator. This search was performed in February 2016, and repeated in October 2016. A protocol was not published in advance.

Reviewer LC screened titles and abstracts for relevant studies, prior to review of full texts of articles. At each stage a sample of studies were reviewed independently by DH with any disagreements resolved by consensus or by arbitration with reviewer IS.

Studies were included if they were systematic reviews investigating a correlation between the serum concentration and either the efficacy of the drug, or the frequency/severity of adverse effects for a condition. There was no restriction placed on the type of studies considered within the systematic reviews (e.g. randomised controlled trials, cohort studies, case reports, etc). In addition, there were no restrictions on the medication used, indication(s) for which the medication was used, the number of concurrent medications, co-morbidities of the patients who received the medication, age or gender of the participants. We excluded systematic reviews comparing therapeutic drug monitoring vs clinical monitoring, narrative reviews and cost benefit analyses as these do not generate information that can inform a drug’s therapeutic range.

Reviewer LC extracted data from each of the included systematic reviews. Predefined review characteristics included the drug name, indication, and characteristics of participants and the aim of the review. We assessed whether a predetermined protocol was followed, how outcomes were selected, how relevant literature was identified, recommendations for clinical practice and the techniques used for data synthesis. An AMSTAR assessment was performed on each included systematic review to assess its quality. This tool assesses the domains of study identification and selection, data analysis and bias assessment [7]. These findings were used to form recommendations for conducting a systematic review of therapeutic ranges.

## Results

### Characteristics of included studies

**Fig. 1:**
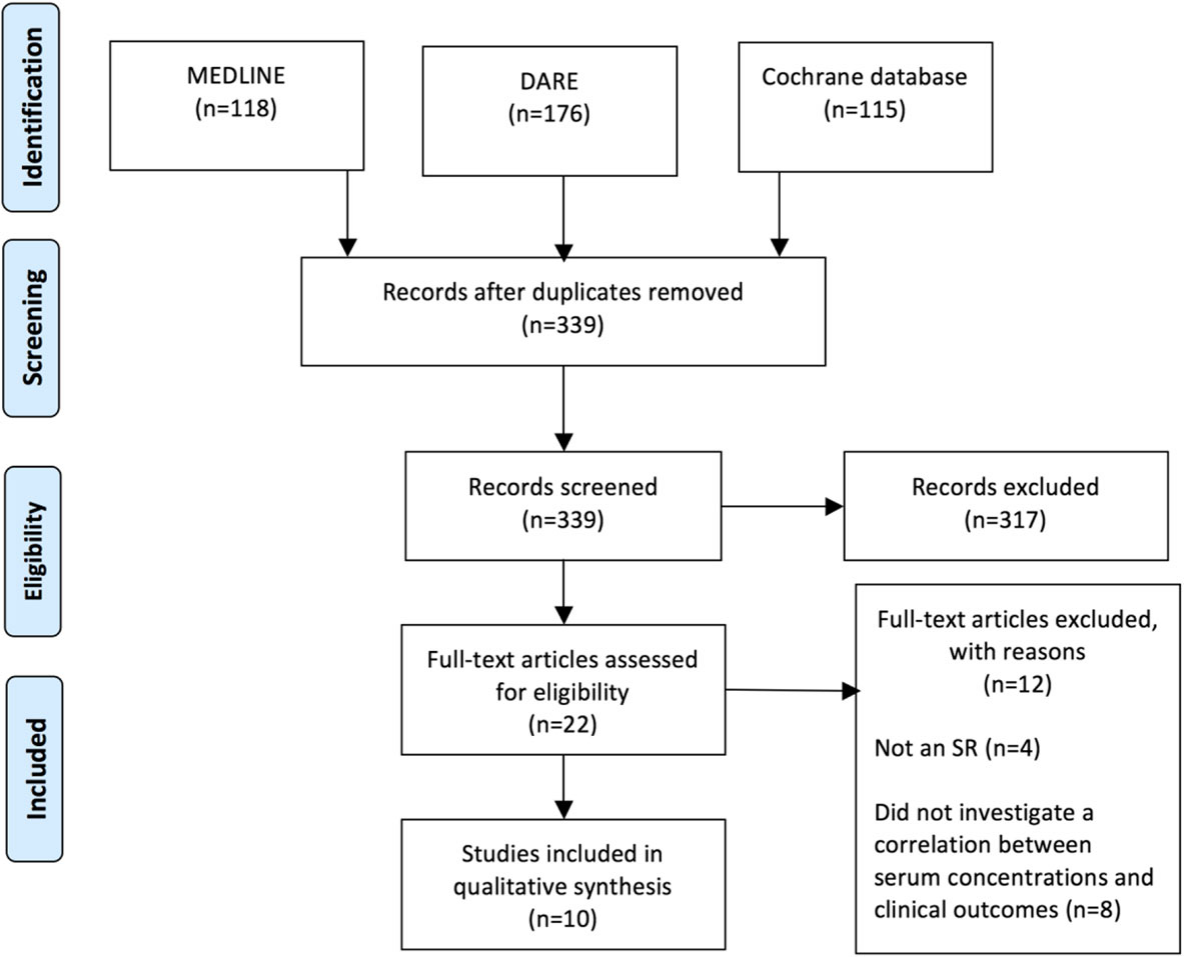
Search results. DARE - Database of Abstracts of Reviews of Effects, SR – Systematic review

The search strategy returned 339 studies after duplicates were removed. After screening titles and abstracts twenty-two full text articles were assessed for eligibility. Of these, eight studies were excluded as they did not investigate a correlation between serum drug level and clinical outcomes and four studies were not systematic reviews (see Fig. 1).

The ten systematic reviews were published from 2006 to 2016 and studied thiopurine metabolites (*n* = 3), mycophenolate mofetil (in post-transplant patients) (*n* = 2), vancomycin trough concentrations (*n* = 1), amisulpride (*n* = 1), aripiprazole (*n* = 1), olanzapine (*n* = 1), and aminophylline (*n* = 1). The systematic reviews encompassed a variety of indications including, inflammatory bowel disease, asthma, Staphylococcous aureus infections, psychiatric disorders and patients receiving kidney, cardiac and liver transplants. All ten systematic reviews investigated the effects of serum concentrations on disease activity, while five systematic reviews also assessed the correlation between serum levels and adverse effects (Table 1).

**Table 1:** Included studies

Author	Drug	Indication	Protocol	Number of studies	Adverse effects	Study design	“drug monitoring” included in search	Information sources	RoB/Quality assessment of studies	Meta analysis
Cooney 2016 [8]	Aminophylline	Asthma	No	12	Yes	No restrictions	No	Medine, CINAHL, Cochrane Central, and Web of Science	Cochrane tool for assessment of risk of bias [35], CASP tool [36]	No
Sparshatt 2009 [10]	Amisulpride	Schizophrenia/schizoaffective disorder	No	10	Yes	No restrictions	No	Embase, Medline and PubMed	None	No
Sparshatt 2010 [14]	Aripirazole	No specific diagnosis	No	8	No	No restrictions	Yes	Embase, Medline, and PubMed	None	No
Knight 2008 [11]	Mycophenolate Mofetil	Transplant patients	No	12	Yes	RCTs, Observational studies	No	Medline, Embase, Cochrane Central, Transplant Library, Clinical trial registries	None	No
Zuk 2009 [15]	Mycophenolate Mofetil	Heart transplant	No	7	No	No restrictions	Yes	Medline Embase	None	No
Bishara 2013 [16]	Olanzapine	Schizophrenia/schizoaffective disorder/bipolar disorder	No	30	Yes	No restrictions	Yes	PubMed, Medline, Embase	None	Yes
Konidari 2014 [9]	Thiopurine	IBD	No	15	Yes	RCTs, Observational studies	Yes	PubMed, Medline, UK national health system database	NICE defined criteria for quality assessment of case series [37]	No
Moreau 2014 [17]	Thiopurine	IBD	Yes	17	No	No restrictions	Yes	Medline, Cochrane Library, DirectScience, and Google Scholar	None^a^	Yes
Osterman 2006 [12]	Thiopurine	IBD	No	12	No	No restrictions	No	Medline, PubMed Plus	None	Yes
Prybylski 2015 [13]	Vancomycin	*S. aureus* bacteraemia	No	14	No	Observational studies	No	PubMed	None	Yes

*RoB* Risk Of bias, *IBD* Inflammatory Bowel Disease, *RCT* Randomised controlled trial, *CENTRAL* Central Register of Controlled Trials

^a^Moreau et al. used the MOOSE consensus statement to assess reporting quality, but do not report the results of their assessment

### Study participants

Of the included systematic reviews, two examined paediatric populations [8, 9] and four adult popuations [10–13]. The remaining four systematic reviews imposed no age restrictions of participants [14–17].

### AMSTAR assessment

The results of the AMSTAR assessment are shown in Table 2. The majority of systematic reviews performed a comprehensive literature search and appropriate techniques for data synthesis. A tool for assessment of quality/risk of bias was used in only three reviews. The tools used were i) the National Institute for Health and Care Excellence (NICE) defined criteria for the assessment of case series [9], ii) Meta Analyses Of Observational Studies in Epidemiology (MOOSE) consensus statement [17], and iii) Cochrane tool for the assessment of risk of bias [8]. Though these systematic reviews used standardized assessment tools, the results of these assessments were not formulated into conclusions. The remaining systematic reviews used no standardized assessment tool for the appraisal of quality or risk of bias in individual studies. Three reviews assessed the potential of publication bias across studies using the Egger method [12, 13, 17].

**Table 2:** Results of AMSTAR assessment

	Pryblyski 2015	Konidari 2014	Moreu 2014	Sparshatt 2009	Knight 2008	Osterman 2006	Cooney 2016	Sparshatt 2010	Zuk 2009	Bishara 2013
Was an a priori design (protocol) provided?	✗	✗	✓	✗	✗	✗	✗	✗	✗	✗
Was there duplicate study selection and data extraction?	✗	✓	✓	✗	✗	✗	✓	✗	✗	✓
Was a comprehensive literature search performed?	✗	✓	✗	✓	✓	✗	✓	✗	✓	✓
Was the status of publication (i.e. grey literature) used as a criterion?	✗	✓	✗	✗	✓	✗	✓	✗	✗	✓
Was a list of studies (included and excluded) provided?	✗	✓	✓	✗	✗	✗	✓	✓	✗	✓
Were the characteristics of the included studies provided?	✓	✗	✓	✓	✓	✓	✓	✓	✓	✗
Was the scientific quality of the included studies assessed?	✗	✓	✗	✗	✗	✗	✓	✗	✗	✗
Was the scientific quality of the studies used in formulating conclusions?	✗	✗	✗	✗	✗	✗	✗	✗	✗	✗
Were the methods used to combine the findings of the studies appropriate?^a^	✓	✗	✗	✗	✗	✓	✓	✗	✗	✓
Was the likelihood of publication bias assessed?^b^	✓	✗	✓	✗	✗	✓	✗	N/A	N/A	✗
Was a conflict of interest included?	✗	✓	✓	✓	✓	✗	✓	✓	✓	✓

^a^Methods used to combine the findings of studies were deemed inappropriate if no a priori statistical techniques were outlined in the methodology

^b^AMSTAR methodology states systematic reviews with <10 studies included do not to assess publication bias (marked N/A)

### Protocols used

Of the ten included systematic reviews, one followed a predefined protocol [17]. This systematic review followed a generic protocol for the meta-analysis of clinical trials [18]. None of the included reviews followed a specific protocol for systematic reviews of therapeutic ranges.

### Identifying relevant studies

All included systematic reviews involved PubMed/MEDLINE in their search strategies, five studies searched Embase [10, 11, 14–16]. Other literature searches included Google Scholar and DirectScience [17], clinical trials registers [11] and the UK national health system database [9]. Two systematic reviews included a grey literature search via the Cochrane Register of Controlled Trials (CENTRAL) [8, 11].

### Included studies

All included reviews analysed more than one study design. Six systematic reviews placed no restrictions on the included study design [10, 12, 14–17], one systematic review included observational data only [13], and two systematic reviews limited studies to randomised controlled trials, cohort studies and case control studies [9, 11].

### Search terms

Five systematic reviews included the search terms ‘drug monitoring’, [9, 14–17]. Acronyms such as TDM [10] and AUC/MIC [13] were also used. Other terms included “Blood AND monitoring”, “physiologic monitoring”, [9] or “plasma levels” [10]. One systematic review did not outline the exact search terms used in the methodology [11], and two reviews used no terms specifically relating to therapeutic drug monitoring [8, 12].

### Data extraction

For comparing serum drug levels achieved in each systematic review, four reviews extracted an arbitrary threshold level, and measured the beneficial effects of the drug above this threshold [9, 12, 13, 17], and two extracted the data for area under the curve (AUC) or data on single point monitoring [11, 15]. The remaining reviews extracted the mean ± standard deviation of drug level achieved across participants [8, 10, 16]. One review extracted the timing of drug measurement [11], and two reviews performed separate analyses depending on the type of assay used [13, 17].

### Statistical analyses

Of the ten included systematic reviews, four used meta-analyses to investigate the association between low versus high serum drug levels and clinically relevant outcomes (Table 3). One of these reviews assessed the relationship between vancomycin trough levels and treatment failure, defined as a composite endpoint that included mortality and persistent bacteraemia. This review used data from several primary studies, which compared the occurrence of treatment failure in patients with serum levels below 15 mg/l with patients above 15 mg/l. This threshold level was chosen based on a cohort study indicating a two-fold risk of treatment failure when vancomycin levels are <15 mg/l in the treatment of MRSA bacteraemia [19]. This study relied on primary studies using similar threshold levels [13]. Two systematic reviews investigated the relationship between threshold values of tioguanine-6 (TGN-6) and clinical remission in patients with inflammatory bowel disease. In one systematic review, the authors only included studies which compared participants with TGN-6 concentration ≥ 230 pmol/8 × 10^8^^8 RBC with those below, with this figure decided a priori [17]. One study performed a similar analysis, but included studies with different threshold levels in the same meta analysis (either 235 pmol/8 × 10^8^ or 250 pmol/8 × 10^8^) [12]. The fourth systematic review planned a meta analysis investigating the relationship between high and low olanzapine serum concentrations on symptom scores in patients with schizoaffective disorder, however there was insufficient data reported to enable meta analysis [16].

**Table 3:** Meta analyses performed

Study	Intervention	Comparator	Outcome
Pryblyski 2015 [13]	Vancomycin serum levels >15 mg/l	Vancomycin serum levels <15 mg/l	Mortality, persistent bacteraemia
Moreau 2014 [17]	Patients with serum TGN concentrations above 230 pmol/ pmol/8.10^8	Patients with serum TGN concentrations below pmol/8.10^8	Clinical remission of inflammatory bowel disease
Osterman 2006 [12]	Patients with serum TGN concentrations above predetermined threshold	Patients with serum TGN concentrations below predetermined threshold	Clinical remission of inflammatory bowel disease
Osterman 2006 [12]	Patients with active disease	Patients in clinical remission	Serum TGN levels
Bishara 2013 [16]	High serum olanzapine concentrations	Low serum olanzapine concentrations	PANSS

*TGN* tioguanine-6, *PANSS* Positive and negative symptom scale

Six systematic reviews did not perform quantitative data synthesis, and instead data analysis was done descriptively. Four reviews planned no meta analysis a priori in their methodologies, it is not clear in these studies why quantitative data synthesis was not performed [9–11, 15]. One review performed a meta analysis correlating dosage to clinical outcomes, but had insufficient data for a similar analysis correlating plasma concentrations of aripirazole to clinical outcomes [14]. One systematic review planned a meta analysis a priori, but this was deemed inappropriate by the investigators following data extraction due to the heterogeneity of data due to variability in the threshold of drug levels used, and inconsistencies in reporting of drug levels achieved across participants [8].

### Clinical relevance

No included systematic reviews were able to provide a definitive therapeutic range from systematically reviewed evidence. Though a relationship between serum concentration and clinical efficacy was found in three reviews [10–12], this was not translated into definable upper and lower limits of drug concentrations that conferred maximum efficacy and safety. This is because a definable cut off point reflecting efficacy and safety could not be established at the lower and upper limits respectively. One systematic review found that the established therapeutic range was able to predict toxicity but unable to predict clinical benefit in patients receiving thiopurine for IBD [9], and three reviews found a weak association between serum drug concentrations and clinical outcomes [8, 11, 13].

## Discussion

Published systematic reviews correlating serum concentrations of a drug with clinical outcomes are scarce. There were none identified that were able to compare RCTs of different therapeutic ranges, and hence other study designs were used in data synthesis.

A strength of review is that it has identified methodological variations, as well as considerable inconsistencies, in the reporting of existing published systematic reviews of therapeutic ranges. There is clearly a need to improve or standardize methods. A limitation of this review is that it only identifies drugs in which the therapeutic range has been systematically reviewed. There are classes of drugs where there is considerable evidence supporting the therapeutic ranges but no systematic reviews have been undertaken (for example, aminoglycoside antibiotics).

We have developed recommendations for future reviewers examining therapeutic ranges based on these findings.

### Determining the scope and question of the review

Most of our included systematic reviews investigated the effect of serum concentrations of a particular drug, for a particular indication, in a particular population. Similar to typical systematic reviews, the research question should be specified a priori. The reviewers should report whether their review is focussed on the lower limit of the therapeutic range, the upper limit, or both; and the population, indication, chronicity and route of the drug. It is important to consider these when deciding the review question as drugs may be used for more than one indication, and in groups of patients with differing characteristics. If the medicine has more than one indication, an appropriate range for each use should be explored as the optimal therapeutic range may vary with age, population and indication. For example, the plasma salicyclate range for the treatment of inflammatory conditions is considerably greater than that required for analgesia [20].

### Outcomes

Selected outcomes must reflect beneficial and harmful effects of the drug that are relevant to clinical practice, in context of the population of interest. For example, focussing on surrogate physiological outcomes such as improvements in Forced Expiratory Volume in One Second (FEV_1_) in asthma [21] and electroencephalogram (EEG) measurements in epilepsy [3] may not reflect whether the drug has a beneficial clinical effect at a given concentration.

It is unclear how review outcomes were selected in most systematic reviews. One way of identifying important outcomes is to see whether there is an established core outcome set, which should be measured and reported in all clinical trials in a given condition [22]. The Core Outcome Measures in Effectiveness Trials (COMET) Initiative contains a database of existing core outcome sets [23], however this does not comprehensively cover all possible harmful effects of drugs. A framework of outcomes has been developed [24], which may help decide which are most important and relevant for the particular topic under review. In some situations, patients have been involved in identifying which outcomes are directly relevant to them [25], and if such studies are available they should be considered when identifying appropriate outcomes for a systematic review.

With regards to adverse effects of drugs, at particular serum concentrations, it is important to consider whether the scope of the review should be broadened to include outcomes in wider populations and across other indications [26]. This will not only be determined by the time and resources available, but also whether the pharmacological properties of the drug, and the susceptibility of the patient to adverse effects, varies between groups. One example where this may be particularly relevant is in preterm neonates, who are uniquely prone to long term side effects which may not be seen in other populations, for example cerebral palsy from steroid use [27–29], and problems with cardiovascular adaptation to extra-uterine life from non-steroidal anti-inflammatory drugs [30].

### Included studies

Reviewers must balance the need to capture all relevant data with the time and resources available. Many therapeutic ranges are under researched, and few primary studies specifically aim to investigate the therapeutic range of a drug. Studies of most interest will be those that report both a measure of serum drug concentration and at least one of the selected clinical outcomes. Authors may consider the levels of available evidence they may find prior to searching for relevant studies. Categorising studies in this way helps systematically grade the available evidence. This avoids placing inappropriate weight on studies that are methodologically less robust when formulating conclusions.

The most useful studies are well designed randomised controlled trials (RCTs) comparing two or more therapeutic ranges for the same drug, and reporting clinically relevant outcomes. Such studies have been conducted to examine serum concentrations of lithium in adults with bipolar disorder for prophylaxis of mania [31] and serum concentrations of teicoplanin in adults who are critically ill with gram negative infections [32].

In practice, trials directly comparing therapeutic ranges are uncommon, and we propose that the next level of evidence will incorporate either observational data or indirect comparisons between clinical trials such as RCTs comparing drug to placebo/other treatment or observational studies.

Observational data may be obtained from cohort studies, case-control studies, or case series. Indirect evidence may be gained from identifying RCTs that have compared the drug against a common comparator (likely placebo), and then analysing whether studies in which a higher serum concentration was achieved demonstrated better effects on beneficial and harmful outcomes.

### Search terms

Combinations of indexing terms such as Medical subject headings (MeSH) and textwords should be used by reviewers to form a search strategy that maximizes sensitivity whilst minimizing specificity whilst keeping the number of records to sift manageable within the logistical constraints of the review. If indirect evidence is considered for inclusion, the search strategy must be sufficiently sensitive to return studies that are not necessarily ‘labelled’ as investigations into the therapeutic range of a drug (e.g. RCTs comparing drug to placebo). As Information on therapeutic ranges may be presented using variable terminology, MeSH terms such as “Drug-Related Side Effects and Adverse Reactions”, “Drug monitoring”, “Pharmacovigilance”, “Adverse Drug Reaction Reporting Systems”, “Monitoring, Physiologic”, “Clinical Pharmacy Information Systems” may lead to inclusion of relevant studies in MEDLINE and other relevant indexing terms will be necessary in other databases.

### Quality/risk of bias assessment

An assessment of quality allows the reviewers to present the degree to which the results in the available literature are likely to be valid and robust, and whether conclusions that impact on clinical practice should be implemented. There are specific considerations when conducting a systematic review of therapeutic ranges.

There are a large number of critical appraisal tools for different study designs, each with varying emphasis on different aspects of quality, although many lack information on how they were developed [33]. These include the Critical Appraisal Skills Programme (CASP) tools, the Graphical Appraisal Tool for Epidemiology (GATE) and the Case Reports (CARE) tool. These tools were not devised for the appraisal of therapeutic drug monitoring studies specifically, and therefore they may not address specific concerns when assessing the quality of included studies in reviews investigating the optimum therapeutic range of a drug.

Risk of bias in RCTs can be conducted using the Cochrane risk of bias (RoB) tool available from http://handbook.cochrane.org/chapter_8/8_assessing_risk_of_bias_in_included_studies.htm. This tool was developed for systematic reviews investigating the efficacy of an intervention, and allows a systemic and consistent approach to all included RCTs. Though this tool allows a systemic and consistent approach, it is limited by its inter-assessor variability, and its tendency to judge older studies as a higher risk of bias. Risk of bias in non-randomised studies can be assessed using the ROBINS-I tool [34].

Although a specific systematic assessment tool for TDM studies is yet to be developed, we suggest the following considerations to be integrated into the quality assessment of included studies. Different assays vary in accuracy and this can lead to misleading measurements of serum concentrations and reviewers should consider the appropriateness of the methods for determining serum drug concentrations in individual participants. Furthermore, drug clearance is a dynamic process and the time period between drug administration and serum measurement should be consistent between participants. Selected outcomes should adequately represent harm and benefit, and techniques for measuring adverse effects should be decided a priori as ad hoc measurement of adverse effects carries risk of selective outcome reporting which could misinform the upper limit of a therapeutic range.

### Statistical analysis

Data synthesis in systematic reviews of therapeutic ranges will involve a comparison of outcomes between participants who demonstrate differing serum levels of a drug. Studies vary in how they summarize the serum levels, and this will impact the approach to meta-analysis. For example, studies may compare mean serum levels between those responding to the drug and those not (or experiencing an adverse event or not) (continuous data), in which case a meta-analysis of differences in means can be undertaken to provide a pooled estimate of the difference. Alternatively, studies may compare numbers responding and not responding to a drug (or experiencing an adverse event or not) between those with serum levels below and above a particular threshold (dichotomous data). In such studies it would be common to report the relative risk or odds ratio of the event of interest, in which case a meta-analysis of the effect estimates can be undertaken to provide a pooled effect estimate. In any case, it is important to assess for heterogeneity between studies, and utilising a random effects approach to meta-analysis is recommended where the heterogeneity is found to be large. Potential sources of heterogeneity can also be explored by subgroup analyses and, where possible, meta-regression.

It is important to note that conducting a meta-analysis may not always be possible within a systematic review, and should only be undertaken where there are a sufficient number of studies with comparable outcomes and exposure groups. For example, where patients are grouped according to a drug level threshold, it may only be appropriate to meta-analyse studies which utilize the same, or at least similar, threshold, and some thought would need to be given as to what range of thresholds would be considered similar.

Where a meta-analysis is not appropriate, a descriptive analysis should be provided of the included studies’ findings.

### Interpreting the results of the review

In reviews appraising the evidence around a standard therapeutic range used in practice, the results may have four possible implications. The range may be appropriate, the upper or lower limit should be amended in light of the review results, or there is no evidence on which to make any recommendations (Table 4).

**Table 4:** Clinical implications of systematic reviews of therapeutic ranges

Clinical improvement (lower limit)	Suspected adverse effects (upper limit)	Clinical implication
Better in ‘therapeutic’ participants compared to subtherapeutic	Higher frequency or severity in supratherapeutic participants compared with therapeutic	No change to clinical practice, therapeutic range is correct
Better in ‘therapeutic’ participants compared to subtherapeutic	No difference between therapeutic and supratherapeutic participants	Clinicians should aim serum levels above the lower limit. Additional studies to determine accurate upper range may be required
No difference between therapeutic and subtherapeutic participants	Higher in supratherapeutic participants compared with therapeutic	Clinicians should aim for serum levels below the upper limit. Additional studies to determine the lower range may be required
No difference between therapeutic and subtherapeutic participants	No difference between therapeutic and supratherapeutic participants	Existing therapeutic range not related to outcomes of interest. Additional studies to determine optimal therapeutic range and/or if TDM beneficial for this drug/indication/population

The quality of the evidence around the recommendations of the review should be presented, in context of a summary of the population, indication, and outcomes included in the review.

## Conclusion

Evidence based therapeutic ranges have the potential to improve TDM practice. Current systematic reviews investigating therapeutic ranges have highly variable methodologies and there is no consensus of best practice when undertaking systematic reviews in this field. Consistency in the searching, selection and appraisal of studies is necessary if these reviews are to inform prescribing practice. These recommendations meet a need not addressed by standard protocols.
